# In Vitro Selection of Probiotics, Prebiotics, and Antioxidants to Develop an Innovative Synbiotic (NatuREN G) and Testing Its Effect in Reducing Uremic Toxins in Fecal Batches from CKD Patients

**DOI:** 10.3390/microorganisms9061316

**Published:** 2021-06-17

**Authors:** Mirco Vacca, Giuseppe Celano, Marcello Salvatore Lenucci, Sergio Fontana, Flavia Maria la Forgia, Fabio Minervini, Aurelia Scarano, Angelo Santino, Giuseppe Dalfino, Loreto Gesualdo, Maria De Angelis

**Affiliations:** 1 Department of Soil Plant and Food Sciences, University of Bari, 70126 Bari, Italy; mirco.vacca@uniba.it (M.V.); fabio.minervini@uniba.it (F.M.); maria.deangelis@uniba.it (M.D.A.); 2 Department of Biological and Environmental Science and Technologies, University of Salento, 73100 Lecce, Italy; marcello.lenucci@unisalento.it; 3 Farmalabor srl, 76012 Canosa di Puglia, Italy; s.fontana@farmalabor.it (S.F.); f.laforgia@farmalabor.it (F.M.l.F.); 4 Unit of Lecce, Institute of Sciences of Food Production C.N.R., 73100 Lecce, Italy; aurelia.scarano@ispa.cnr.it (A.S.); angelo.santino@ispa.cnr.it (A.S.); 5 National Institute of Gastroenterology “S. de Bellis” Research Hospital, 70013 Castellana Grotte, Italy; giuseppe.dalfino@irccsdebellis.it; 6 Nephrology, Dialysis and Transplantation Unit, Department of Emergency and Organ Transplantation, “Aldo Moro” University, 70124 Bari, Italy; loreto.gesualdo@uniba.it

**Keywords:** probiotics, prebiotics, antioxidants, microbiota, synbiotic, fecal batches, chronic kidney disease

## Abstract

We aimed to develop an innovative synbiotic formulation for use in reducing dysbiosis, uremic toxins (e.g., *p*-cresol and indoxyl sulfate), and, consequently, the pathognomonic features of patients with chronic kidney disease (CKD). Twenty-five probiotic strains, belonging to lactobacilli and *Bifidobacterium*, were tested for their ability to grow in co-culture with different vegetable (pomegranate, tomato, and grapes) sources of antioxidants and prebiotics (inulin, fructo-oligosaccharides, and β-glucans). Probiotics were selected based on the acidification rates and viable cell counts. Inulin and fructo-oligosaccharides reported the best prebiotic activity, while a pomegranate seed extract was initially chosen as antioxidant source. The investigation was also conducted in fecal batches from healthy and CKD subjects, on which metabolomic analyses (profiling volatile organic compounds and total free amino acids) were conducted. Two out of twenty-five probiotics were finally selected. After the stability tests, the selective innovative synbiotic formulation (named NatuREN G) comprised *Bifidobacterium animalis* BLC1, *Lacticaseibacillus casei* LC4P1, fructo-oligosaccharides, inulin, quercetin, resveratrol, and proanthocyanidins. Finally, NatuREN G was evaluated on fecal batches collected from CKD in which modified the viable cell densities of some cultivable bacterial patterns, increased the concentration of acetic acid and decane, while reduced the concentration of nonanoic acid, dimethyl trisulfide, and indoxyl sulfate.

## 1. Introduction

Chronic kidney disease (CKD) refers to damaged kidneys with decreased capability to filter blood. CKD can evolve into end-stage-renal disease (ESRD), where the kidneys do not work, necessitating dialysis [1,2]. An altered kidney functionality determines an excess of waste metabolites that, accumulating in blood, causes other health problems, such as heart disease and stroke [3]. The incidence of CKD is growing worldwide, becoming a widely recognized problem for public health. The general increase in the average age of the population and the decrease in the mortality of patients at risk of renal failure are among the main factors linked to the increased incidence of CKD. Hence, due to the increasing costs for the management of CKD and ESRD patients, researchers are searching for alternative strategies to treat CKD patients.

In the last years, increasing evidence has highlighted that a bidirectional relationship, defined as the renal–gut axis, exists between gut microbes and nephropathy [4,5]. In CKD, renal damage determines an accumulation of urea and nitrogenous compounds in both blood and intestinal lumen [6]. Therefore, urea and its derivatives (i.e., ammonia and other nitrogenous compounds) cause significant deviations in the optimal pH range of the gut, and both urea and intestinal pH strongly favor the proliferation of proteolytic bacteria [7,8]. From this point of view, the genomic potential of specific bacterial patterns (e.g., Proteobacteria and relative sub-taxa, such as *Enterobacteriaceae*) determines the production of secondary metabolites, starting from the overabundant nitrogen compounds found in the intestinal lumen of nephropathic subjects [8]. Those secondary metabolites, known as uremic toxins (e.g., trimethylamine-*N*-oxide, TMAO, indoxyl sulfate, IS, and *p*-cresyl sulfate (*p*CS)), cause a worsening of renal function and, therefore, the establishment of a vicious cycle along the renal–gut axis [9,10].

In this context, some novel strategies to treat nephropathy are based on either nutritional therapy (e.g., low- or very-low-protein diets) [11,12,13] or the use of probiotics and prebiotics, alone or in combination (i.e., synbiotics) [14,15,16,17]. The common aim of these alternative strategies is to reduce the typical dysbiosis and symptoms affecting subjects with low kidney functionality, shifting from the dominance of proteolytic gut microbiota to saccharolytic. These strategies also aim to delay ESRD onset and, thus, the need for dialysis. The shift from proteolytic to saccharolytic metabolism firstly leads to a reduction in the production and accumulation of uremic toxins. Additionally, the production of short-chain fatty acids (SCFAs) by saccharolytic bacteria has also been largely recognized as beneficial [18,19] in CKD patients [17,20,21].

Nowadays, a large body of evidence is available about the effects caused by the administration of probiotics and synbiotics to CKD patients [22,23,24,25,26]. The framework of symptomatology linked to CKD is widely recognized, and bacterial patterns and relative metabolites that mark the nephropathy are known. However, one of the major limitations of innovative formulations is the minimal evidence for their efficacy under specific conditions before being applied in vivo. In some cases, probiotics may not be able to survive in specific conditions and/or environments. Consequently, in our opinion, it is necessary to start from in vitro evaluations that simulate the conditions encountered by probiotics and synbiotics used for treating CKD.

Therefore, before proceeding with in vivo evaluation, and to develop an innovative synbiotic with a prospective application in CKD patients, we aimed to select the best combination between probiotics, prebiotics, and antioxidants from vegetables and vegetable extracts. Once found, this combination was used in an innovative formulation aiming to assess, through testing of fecal samples collected from nephropathic patients, its potential effect on affecting the gut microbiota and the concentration of uremic toxins.

## 2. Materials and Methods

### 2.1. Microorganisms and Culture Conditions

Twenty-four probiotic strains, belonging to *Bifidobacterium breve* (one strain), *Bifidobacterium animalis* subsp. *lactis* (two strains), *Lactobacillus* (*L.*) *acidophilus* (two), *Lacticaseibacillus* (*Lc*.) *casei* (three), *L. delbrueckii* subsp. *bulgaricus* (one), *Lc*. *paracasei* (one), *Lactiplantibacillus* (*Lp*.) *plantarum* (nine), *Limosilactobacillus* (*Ls*.) *reuteri* (two), and *Lc*. *rhamnosus* (three), were obtained from either commercially available formulations or animal and human feces. In the latter case, the strains belonged to the Culture Collection of the University of Bari. In addition to pure cultures of probiotic strains, the commercial formulation Synbio 100 (Sacco s.r.l., Cadorago, Italy), which includes *Lc*. *rhamnosus* and *Lc*. *paracasei* subsp. *paracasei* in a 1:1 ratio was used in this study. Strains were propagated in MRS broth (Oxoid Ltd., Basingstoke, Hampshire, England, UK) for 24 h at 37 °C, except for *Lp*. *plantarum* 12A, *Lc*. *casei* LC4P1 and BGP93, and *Lc*. *paracasei* 14A, which were cultured at 30 °C.

### 2.2. Chemical Characterization of Food Matrices

Whole tomato fruits or grape skin were frozen in liquid nitrogen, freeze-dried, and finely ground, before extraction with 80% methanol at 4 °C overnight. Extracts were centrifuged at 5000× *g* for 20 min at 4° C and supernatants were collected and filtered through a 0.22 μm filter. Main polyphenols classes were characterized through HPLC, as reported by Scarano et al. [27]. All analyses were performed in triplicate (sampling replicas) for each extract.

Additionally, four different varieties of pomegranate (*Punica granatum* L.; Kamel, Emek, Ako, and Wonderful one) were characterized. Briefly, after pomegranate fruit collection, the relative juice was extracted and analyzed for moisture, ash, total soluble solids, and pH. In addition, total carbohydrates, polysaccharides, pectins, starch, sugars, organic acids, ascorbic acid (AsA), and dehydroascorbic acid (DHA) were determined. Lastly, the pomegranate juices were characterized for their antioxidant activity through the determination of soluble and insoluble-bound phenols, flavonoids, proanthocyanidins, and anthocyanins. The detailed methods are reported in Appendix A.

### 2.3. Evaluation of Effects Exerted by Antioxidants and Prebiotics on Probiotics Growth

In order to evaluate the effects of antioxidants on probiotics growth, all tomato extracts and pomegranate juices were tested. In a subsequent step of the workflow, a commercial pomegranate seed extract (Pom.S.E.) (Farmalabor s.r.l., Canosa di Puglia, Italy), with known high levels of antioxidants and low amount of carbohydrates, was also tested.

All the extracts were added at a concentration of 5 g/L to laboratory-made MRS (artisanal MRS (artMRS)), which contained peptone (10 g/L), meat extract (10 g/L), yeast extract (5 g/L), glucose (2 g/L), di-potassium phosphate (2 g/L), sodium acetate (5 g/L), tri-ammonium citrate (2 g/L), magnesium sulfate (0.2 g/L), manganese sulfate (0.05 g/L), and Tween 80 (polysorbate, 1 mL/L). After sterilization at 121 °C for 15 min, probiotics were added at a cell density of 7 log CFU/mL and incubated at 37 °C for 24 h. The pH variation (ΔpH) and bacterial cell counts on MRS agar medium (Oxoid Ltd., Basingstoke, Hampshire, England, UK) were determined.

The same workflow was adopted for prebiotics, i.e., inulin, fructo-oligosaccharides (FOS), and β-glucans. Due to the possible negative or positive effects of food antioxidants on the growth of different intestinal microorganisms, including probiotics, microbiological tests were conducted to select prebiotics even in the presence of antioxidants. Prebiotics were added, singularly or in combination, at a concentration of 5 g/L in art-MRS at a 1:1 ratio (each 2.5 g/L) when combined. After sterilization at 121 °C for 15 min, probiotics were added at a cell density of 7 log CFU/mL and incubated at 37 °C for 24 h. ΔpH and bacterial cell counts were determined, as above.

### 2.4. Fecal Media from Healthy Subjects and Chronic Kidney Disease Patients

To evaluate the capability of selected probiotics to grow in co-culture under conditions simulating the intestinal ecosystem, fecal extracts were used as model media [28]. The fecal media consisted of pooled fecal extracts from five healthy subjects (HC) or five nephropathic patients (CKD; stage IIIb–IV). Briefly, fecal extracts were obtained from human feces (HC or CKD) freshly collected in a sterile stool container. Then, containers were opened under a sterile hood, diluted 1:10 with distilled water, and homogenized with a stomacher (Bag Mixer, Interscience International, Roubaix, France) for 3 min. The obtained suspension was then centrifuged at 14,000 rpm for 15 min. To the supernatant, we added di-potassium phosphate (2 g/L), sodium acetate (5 g/L), tri-ammonium citrate (2 g/L), magnesium sulfate (0.2 g/L), manganese sulfate (0.05 g/L), Tween 80 (polysorbate, 1 mL/L), and glucose (2 g/L, instead of 20 g/L usually used for probiotics growth), and it was sterilized at 121 °C for 15 min. The obtained fecal media were singly inoculated with the selected probiotics at a cell density of 7 log CFU/mL and incubated at 37 °C for 24 h.

### 2.5. Amino Acids Extraction and Detection

Total free amino acids (FAAs) in water-soluble extracts of fecal media were determined using a Biochrom 30 series Amino Acid Analyzer (Biochrom Ltd., Cambridge Science Park, Waterbeach Cambridge, UK), equipped with a sodium cation-exchange column (20 by 0.46 cm, inner diameter), as previously described [29]. The analysis was performed after 24 h of probiotic growth in fecal media obtained from pooled and sterilized feces of healthy subjects (HC) or chronic kidney disease patients (CKD).

### 2.6. Short Chain Fatty Acids Detection

Short chain fatty acids were detected in fecal media using gas chromatography-mass spectrometry (GC-MS) according to Dixon et al. [30]. Determination was performed using the same samples indicated in Section 2.5. After preconditioning (according to the manufacturer’s instructions) a polydimethylsiloxane/divinylbenzene (PDMS/DVB) fiber (65 μm), a manual solid-phase micro-extraction (SPME) holder (Supelco Inc., Bellefonte, PA, USA) was used. Before headspace sampling, the fiber was exposed to the gas chromatography (GC) inlet for 1 h for thermal desorption at 250 °C. Three grams of sample were placed into 10 mL glass vials, and 10 μL of 4-methyl-2-pentanol (final concentration 33 mg/L) was added as the internal standard. Samples were then equilibrated for 10 min at 40 °C. The SPME fiber was exposed to each sample for 40 min. Equilibration and absorption phases were conducted under stirring. The fiber was then inserted into the injection port of the gas chromatograph for 10 min of sample desorption. GC-mass spectrometry (MS) analyses were performed with an Agilent 7890A gas chromatograph (Agilent Technologies, Palo Alto, CA, USA) coupled to an Agilent 5975C mass selective detector, operating in electron impact mode (ionization voltage, 70 eV). A Supelcowax 10 capillary column (length, 60 m; inner diameter, 0.32 mm; Supelco, Bellefonte, PA, USA) was used. The temperature program was 50 °C for 1 min, followed by an increase at a rate of 4.5 °C/min to 65 °C, an increase at a rate of 10 °C/min to 230 °C, and then holding at 230 °C for 25 min [31]. The injector, interface, and ion source temperatures were kept at 250, 250, and 230 °C, respectively. The mass-to-charge ratio interval was 30 to 350 Da and spectra were acquired at a rate of 2.9 scans per second. Injection was carried out in splitless mode, and helium (flow rate = 1 mL/min) was used as the carrier gas. Molecules were identified based on the comparison of their retention times with those of pure compounds (Sigma-Aldrich, Milan, Italy). Identities were confirmed by searching mass spectra in the available databases (NIST, version 2005; Wiley, version 1996). Quantitative data for the identified compounds were obtained by interpolation of the relative area versus the internal standard area. All the GC-MS raw files were converted into netCDF format via Chemstation (Agilent Technologies, Santa Clara, CA, USA) and subsequently processed through the XCMS toolbox (http://metlin.scripps.edu/download/; accessed date: 1 December 2020). XCMS software allows for automatic and simultaneous retention time alignment, matched filtration, peak detection, and peak matching. GC-MS/SPME data were organized in matrices for subsequent statistical analysis.

### 2.7. In Vitro Effects of the Innovative Synbiotic Formulation on the Fecal Microbiota of HC and CKD Subjects

The above-described HC and CKD fecal media were inoculated with 1% (*w*/*w*) of fresh feces from a healthy volunteer or a CKD patient, separately, to constitute the fecal batches. Feces obtained from the two volunteers were collected in sterile stool containers filled over 4/5, with the aim of reducing the headspace. Then, fresh fecal samples were processed within 6 h from the collection, according to the guidelines used for fecal microbiota transplantation [32]. To both fecal batches (HC and CKD), we also added the developed innovative synbiotic formulation and incubated the batches at 37 °C for 24 h under anaerobic conditions. The cultivable bacteria were enumerated before and after incubation. Three independent determinations were carried out for each pool, as previously described [29]. Ten grams of fecal batches was homogenized with 40 mL of sterilized physiological solution (0.9% of sodium chloride (*v*/*v*)). Diluted fecal batches were inoculated in plates containing the following selective media: plate count agar (total facultative aerobes and anaerobes); MRS agar (lactic acid bacteria); Bifidobacterium agar modified (*Bifidobacteria*) (Becton Dickinson France SA, Le Pont de Claix, France); Mannitol salt agar (staphylococci); Wilkins–Chalgren anaerobe agar (total anaerobes); Wilkins–Chalgren anaerobe agar plus GN selective supplements and defibrinated sheep blood (*Bacteroides*, *Porphyromonas*, and *Prevotella*); MacConkey agar No2 (*Enterobacteriaceae*); Rogosa agar plus 1.32 mL/L of glacial acetic acid (lactobacilli); GSP agar (Fluka) plus penicillin-G (100,000 IU/L) (*Pseudomonas*, *Aeromonas*); Slanetz and Bartley (enterococci). Excluding both Bifidobacterium modified agar and GSP agar, all media were purchased from Oxoid.

### 2.8. Uremic Toxins Detection

Concentrations of uremic toxins were determined on two samples obtained from each of the two fecal batches (HC and CKD), to which we added the developed innovative synbiotic. The baseline values (t0) were determined immediately after the addition of fresh feces to fecal media. The uremic toxins amount was also evaluated after incubation at 37 °C (t24). Concentrations of uremic toxins were determined through nuclear magnetic resonance (NMR) analysis, according to Beaumont et al. [33]. Briefly, 70 mg of each sample was homogenized with 1.3 mL NMR buffer for 5 min; the homogenate was centrifuged (13,000× *g* for 10 min at 4 °C), and the supernatant transferred into 5 mm tubes for NMR spectroscopy. After processing, spectra were digitalized and imported in MATLAB (version 2014b) to calculate the uremic toxins concentration.

### 2.9. Statistical Analyses

Data were subjected to one-way ANOVA; a paired comparison of treatment means was carried out by Tukey’s procedure, with *p* < 0.05 indicating statistically significant differences, using the statistical software Statistica for Windows (Statistica Software 7.0, Palo Alto, CA, USA).

## 3. Results

### 3.1. Characterization of Food Matrices Rich in Antioxidants

The four varieties of tomato (wild type (WT), Indigo, ResTom, and Bronze) used in the present work were previously characterized for their polyphenol content (flavonoids, stilbenoids, and anthocyanins) and their anti-inflammatory effects in colitis mouse models [27]. Among the varieties, ResTom is rich in stilbenoids, Indigo in flavonoids and anthocyanins, and Bronze contains a remarkable amount of all three polyphenols. Compared with the WT, the presence of high levels of polyphenols in all the transgenic varieties (Indigo, ResTom, and Bronze) agree with the antioxidant activity of the hydrophilic fractions extracted from ripe fruits, with Bronze showing the highest activity (Supplementary Table S1).

The results of chemical characterization of pomegranate juices (Kamel, Emek, Ako, and Wonderful one) are shown in Supplementary Table S2.

### 3.2. Acidification and Growth of Probiotics in Presence of Antioxidants and Food Matrices

**Figure 1:**
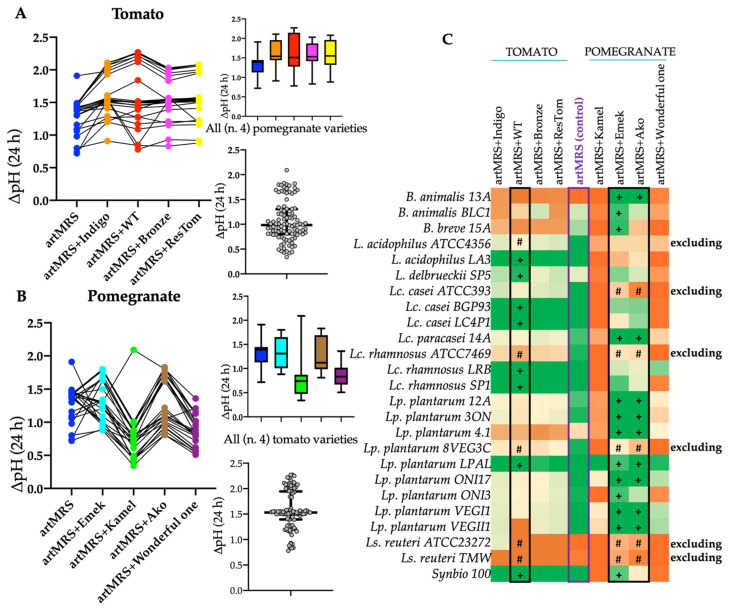
Acidification rate (ΔpH) after 24 h of probiotic growth at 37 °C in artisanal MRS with 2 g/L of glucose (artMRS) used alone, as a control, or supplemented with different antioxidant matrices (5 g/L). (**A**) Evaluation of lyophilized tomato extract from four different varieties, specifically Indigo (artMRS + Indigo), wild type (artMRS + WT), Bronze (artMRS + Bronze), and ResTom (artMRS + ResTom). (**B**) Evaluation of pomegranate juices of four different varieties, specifically Kamel (artMRS + Kamel), Emek (artMRS + Emek), Ako (artMRS + Ako), and Wonderful one (artMRS + Wonderful one). (**C**) Heatmap of the ΔpH values found in media supplemented with lyophilized tomato extracts and pomegranate juices after 24 h of probiotics growth; values range from dark orange (lower ΔpH values, <25th percentile) to dark green (higher ΔpH values, >75th percentile), with light yellow corresponding to intermediate ΔpH values (50th percentile). Data are presented as the average of at least one biological triplicate ± standard deviation (SD). Inside the heatmap, # indicates that the tested condition determined the exclusion of the probiotics, whereas + indicates that the tested condition determined the selection of the probiotics.

To identify the antioxidant(s) to include in our innovative synbiotic formulation, freeze-dried tomato and pomegranate juice extracts were tested. Compared with the control (artMRS), only *B. animalis* BLC1 and *Ls. reuteri* ATCC23272 showed a higher ΔpH when grown in artMRS + Bronze or artMRS + Indigo, respectively (Figure 1C). The highest ΔpH values were found in artMRS + WT tomato extracts (Figure 1A), where 8 out of 25 tested probiotics showed the best acidification rate (Figure 1C). Among the four pomegranate varieties, the highest acidification (ΔpH) was found for probiotics cultured in media with juice from either Ako or Emek (Figure 1B,C).

Due to the low acidification observed when cultured in the presence of antioxidants, *L. acidophilus* ATCC4356, *Lc. casei* ATCC393, *Lc. rhamnosus* ATCC7469, *Ls. reuteri* ATCC23272 and TMW, and *Lp. plantarum* 8VEG3C were excluded from further analyses. In agreement with acidification, the remaining 19 probiotics were able to grow in the presence of antioxidants (data not shown).

**Figure 2:**
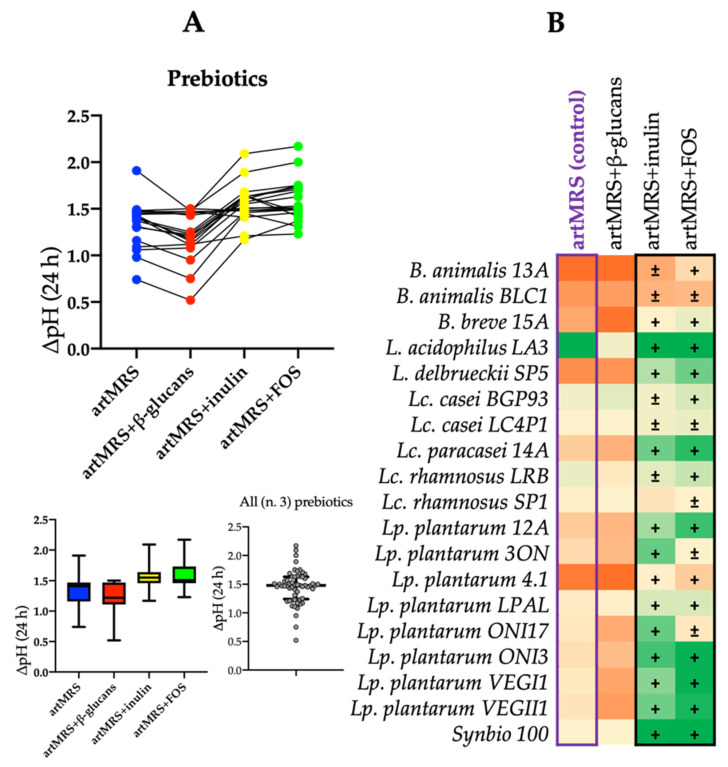
(**A**) Acidification rate (ΔpH) after 24 h of probiotic growth at 37 °C in artisanal MRS with 2 g/L of glucose (artMRS) used alone as a control or supplemented with prebiotics (5 g/L), specifically β-glucans (artMRS + β-glucans), inulin (artMRS + inulin), and FOS (artMRS + FOS). (**B**) Heatmap of the ΔpH values found in artMRS supplemented with different prebiotics after 24 h of probiotics growth; values range from dark orange (lower ΔpH values, <25th percentile) to dark green (higher ΔpH values, >75th percentile), whereas light yellow corresponds to intermediate ΔpH values (50th percentile). Data are presented as the average of one biological triplicate ± SD. Inside the heatmap, + indicates a ΔpH higher than the control, whereas ± indicates a ΔpH similar to the control.

The same workflow was also adopted for prebiotics, specifically β-glucans, inulin, and fructo-oligosaccharides (FOS). Compared with the control (artMRS), strains grown on artMRS with inulin or FOS showed similar or higher ΔpH values (Figure 2A).

**Figure 3:**
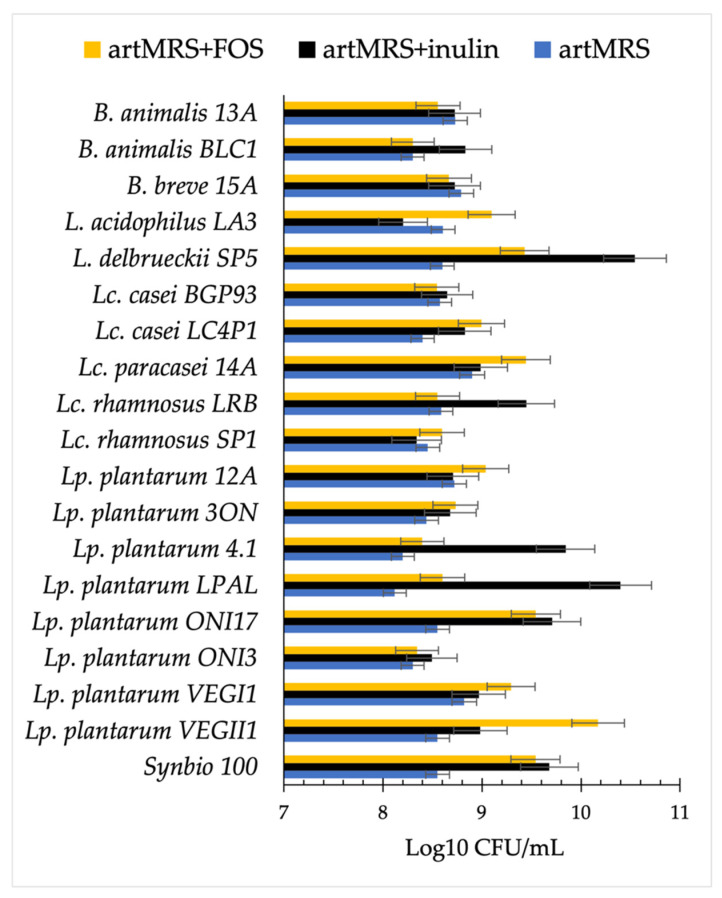
Viable cell count (log CFU/mL) of probiotics after 24 h of growth at 37 °C in artisanal MRS (2 g/L of glucose (artMRS)) with the addition of fructo-oligosaccharides (artMRS + FOS, 5 g/L) or inulin (artMRS + inulin, 5 g/L). Data are presented as the average of one biological triplicate ± SD.

In agreement with the acidification results, all the 19 tested probiotics were able to grow on artMRS with inulin or FOS. Probiotics cultured in the presence of inulin showed, on average, a higher cell density than when cultured in the presence of FOS (Figure 3).

Due to the high carbohydrate concentrations in Ako and Emek pomegranate juice extracts, a further analysis was conducted using a commercial pomegranate seed extract (Pom.S.E.) characterized by a small amount (traces) of carbohydrates. The degree of acidification was evaluated, comparing the control (artMRS) with the following conditions: (i) artMRS + Pom.S.E. (5 g/L), (ii) artMRS + Pom.S.E. (5 g/L) + FOS (5 g/L), and (iii) artMRS+ Pom.S.E. (5 g/L) + FOS (2.5 g/L) + inulin (2.5 g/L). The selected probiotics showed the lowest pH values when grown on medium with added FOS + inulin (Table 1). Compared with the control, 12 of the 19 probiotics showed a difference in pH value that was higher than or equal to 0.3 pH units.

**Figure 4:**
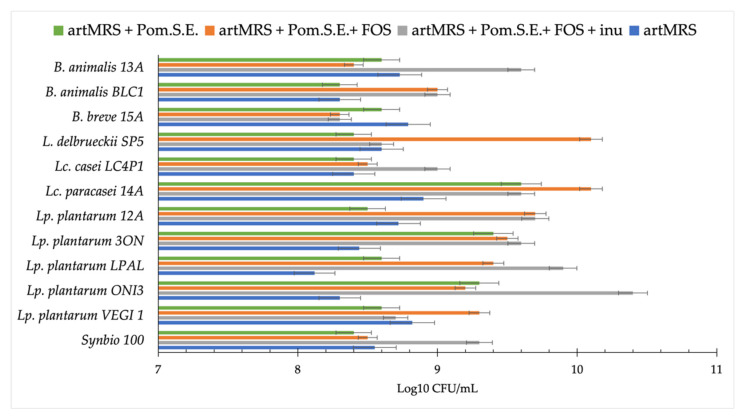
Probiotics viable cell count (log CFU/mL) after 24 h of growth at 37 °C in artisanal MRS (2 g/L of glucose; artMRS) used alone (as a control) or supplemented with pomegranate seed extract (Pom.S.E.; 5 g/L), or Pom.S.E. + fructo-oligosaccharides (FOS) (both 5 g/L), or Pom.S.E. (5 g/L) + FOS (2.5 g/L) + inulin (2.5 g/L). Data are presented as the average of one biological triplicate ± SD.

The viable cell counts of the most acidifying strains (*n* = 12) were determined. After 24 h of incubation, cell density increased (1–3 logarithmic cycles) in all the theses (Figure 4). More specifically, *B. animalis* 13A; *Lc. paracasei* 14A; and *Lp. plantarum* strains LPAL, 12A, and ONI3 showed the highest cell density in the presence of Pom.S.E. and both the prebiotics (FOS and inulin). *L. delbrueckii* SP5; *Lp. plantarum* ONI3, LPAL, and VEGI1; and *Lc. paracasei* 14A showed an increase in the presence of Pom.S.E. and FOS. Based on these results, as well as on the higher acidification observed in the presence of both prebiotics, the condition containing FOS, inulin, and Pom.S.E. was chosen for further analyses.

### 3.3. Acidification and Growth of Probiotics on Fecal Media from Healthy and Chronic Kidney Disease Subjects

Twelve probiotics (*B. breve* 15A; *B. animalis* BLC1 and 13A; *L. delbrueckii* SP5; *Lc. casei* LCP1; *Lc. paracasei* 14A; *Lp. plantarum* LPAL, VEGI 1, ONI3, 3ON, and 12A; and Synbio 100), showing the highest acidification and growth on media containing antioxidant food matrices and prebiotics, were tested for their ability to grow in fecal media.

Fresh fecal samples were collected from five healthy subjects (HC), pooled, and used to produce HC fecal medium. To the fecal medium, we added glucose (2 g/L), dietary fibers (FOS + inulin, each 2.5 g/L), and/or antioxidants (5 g/L). More specifically, the following six experimental treatments were tested:

- The-1: HC fecal media + FOS + inulin.
- The-2: HC fecal media + FOS inulin + Pom.S.E.
- The-3a: HC fecal media + FOS + inulin + tomato extract variety ResTom.
- The-3b: HC fecal media + FOS + inulin + tom. ex. var. Indigo.
- The-3c: HC fecal media + FOS + inulin + tom. ex. var. Bronze.
- The-3d: HC fecal media + FOS + inulin + tom. ex. from WT tomato.

**Figure 5:**
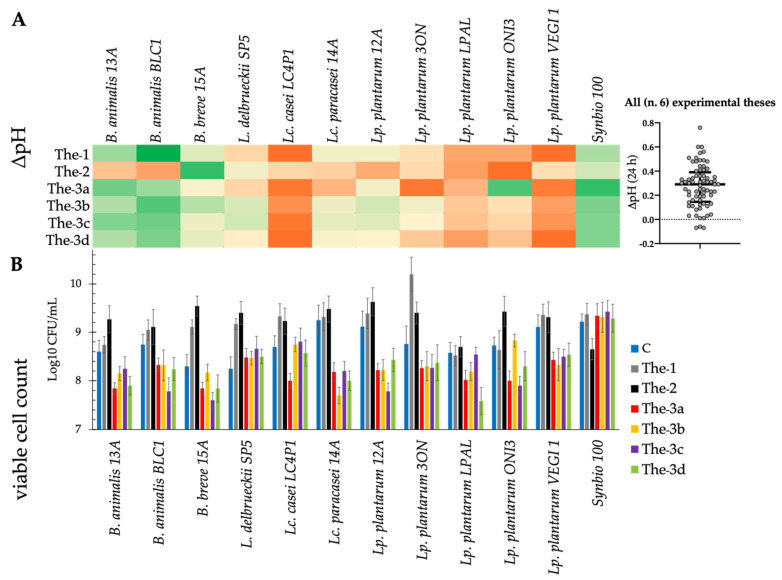
(**A**) Heatmap of the fecal media acidification degree (ΔpH) after 24 h of probiotic growth at 37 °C; values range from dark orange (lower ΔpH values, <25th percentile) to dark green (higher ΔpH values, >75th percentile), while light yellow values correspond to intermediate ΔpH values (50th percentile). All fecal media were obtained using pooled fecal extracts of healthy subjects (HC) adding a low content of glucose (2 g/L). Prebiotic supplements (FOS and inulin) were added at a concentration of 2.5 g/L, while antioxidant ones (Pom.S.E. and tomato extracts) were added at a concentration of 5 g/L. Experimental treatments: The-1, HC fecal media + FOS + inulin; The-2, HC fecal media + FOS + inulin + Pom.S.E.; The-3a, HC fecal media + FOS + inulin + tomato extract var. ResTom; The-3b, HC fecal media + FOS + inulin + tomato extract var. Indigo; The-3c, HC fecal media + FOS + inulin + tomato extract var. Bronze; The-3d, HC fecal media + FOS + inulin + tomato extract from WT tomato. (**B**) Probiotics viable cell count (log CFU/mL) after 24 h of growth at 37 °C in fecal media obtained from pooled fecal extracts of healthy subjects (HC) with the addition of a low content of glucose (2 g/L). Control (C): fecal medium with pooled HC fecal extracts + glucose (2 g/L). The experimental treatments (The-1, The-2, The-3a, The-3b, The-3c, and The-3d) are reported above. Data are presented as the average of one biological triplicate ± SD.

In addition, HC fecal medium with only glucose added was used as the control. Each fecal medium was inoculated with the selected probiotics. After 24 h of incubation, most of the probiotics acidified the fecal media, showing a ΔpH ranging from 0.2 to 0.4 (Figure 5A). *B. animalis* BLC1 showed the highest ΔpH (value: 0.78) when inoculated in The-1, whereas *B. breve* 15A showed the highest ΔpH (value: 0.60) in The-2. Probably due to the production of basic compounds (e.g., NH_3_), some strains showed a pH increase, specifically *Lc. casei* LC4P1 grown The-1 and The-3d (−0.18 and −0.19, respectively) and *Lp. plantarum* ONI3 grown in The-2 (−0.15).

All the probiotics showed an increase (about 1–2 logarithmic cycles) in cell density (Figure 5B). Compared with the control, almost all probiotics showed higher cell count when cultured in the presence of prebiotics alone (The-1) or in combination with pomegranate extract (The-2). The only exception was Synbio 100 in The-2. In presence of tomato extracts (The-3a, The-3b, The-3c, and The-3d), all the strains showed cell density values similar to or lower than that of the control.

Therefore, probiotics growth was assessed in fecal media prepared starting from pooled feces from patients with chronic kidney disease (CKD). The experimental treatments, in which probiotics were singly added, were:

- FM(CKD) + FOS + inu (The-1) = CKD fecal extract + glucose (2 g/L) + FOS (2.5 g/L) + inulin (2.5 g/L);
- FM(CKD) + FOS + inu + Pom.S.E. (The-2) = CKD fecal extract + glucose (2 g/L) + FOS (2.5 g/L) + inulin (2.5 g/L) + pomegranate seed extract (5 g/L).

In addition, a CKD fecal medium, with the addition of glucose only, was used as the control, called FM(CKD).

**Figure 6:**
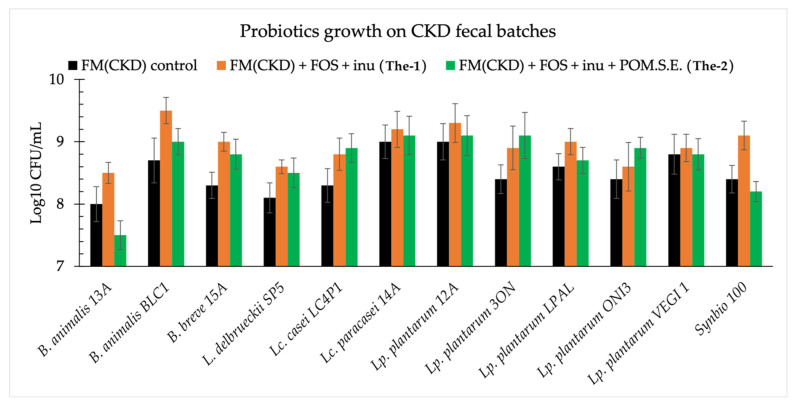
Probiotics growth in fecal media (FM) obtained using feces from chronic kidney disease (CKD) patients with the addition of 2 g/L of glucose, which was named FM(CKD), FM (CKD) + FOS (2.5 g/L) + inulin (2.5 g/L), and FM(CKD) + FOS (2.5 g/L) + inulin (2.5 g/L) + pomegranate seed extract (Pom.S.E.; 5 g/L). Data are presented as the average of one biological triplicate ± SD.

Except for *B. animalis* 13A grown in the presence of both prebiotics and pomegranate, all the probiotics showed an increase of at least one logarithmic cycle. Compared with the control, lower viable cell counts were found for *B. animalis* 13A and Synbio 100 when grown in the presence of prebiotics and pomegranate seed extract. In contrast, under the same conditions, lactobacilli showed a viable cell count that was equal to or higher than that of the control (Figure 6). Therefore, considering the goal of including antioxidants in our innovative synbiotic, the treatment with FOS, inulin, and Pom.S.E. was used to proceed with further analyses.

### 3.4. Concentrations of Total Free Amino Acids

**Figure 7:**
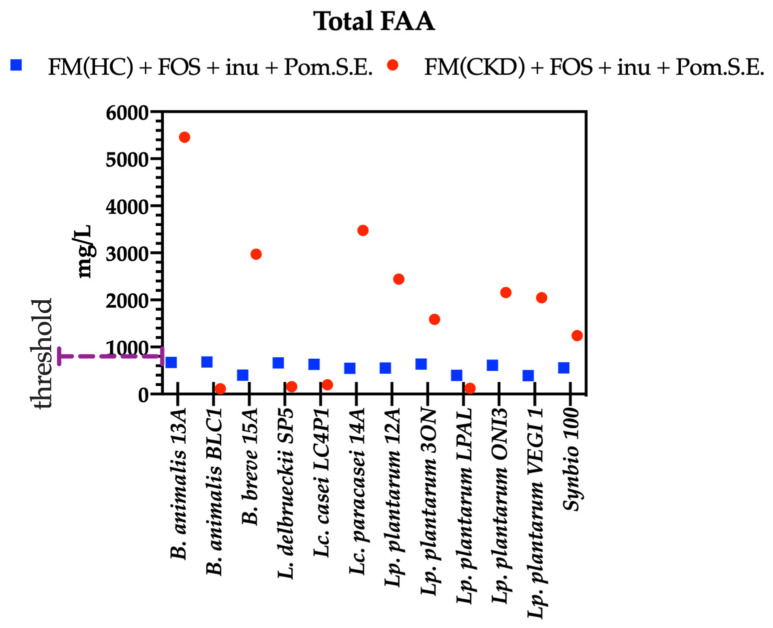
Concentrations of total free amino acids (FAAs, mg/L) after 24 h of probiotics growth in fecal media (FM) obtained from pooled feces of healthy subjects (HC) or chronic kidney disease patients (CKD) + 2 g/L of glucose + FOS (2.5 g/L) + inulin (inu; 2.5 g/L) + pomegranate seed extract (Pom.S.E.; 5 g/L). Data are reported as the average of one technical triplicate.

To develop a synbiotic that is potentially usable in CKD patients, the concentrations of the total free amino acids (total FAAs) of the 12 probiotics were analyzed. Fecal extracts from the fresh feces of HC and CKD subjects were used to constitute two different types of fecal media. These fecal media were supplemented with FOS, inulin, and Pom.S.E. (2.5:2.5:5 g/L, respectively) and singly inoculated with probiotics. After incubation, the concentration of total FAAs was lower than 800 mg/L in the HC fecal media. Conversely, in fecal media obtained from CKD, only 4 (*B. animalis* BLC1, *L. delbrueckii* SP5, *Lc. casei* LC4P1, and *Lp. plantarum* LPAL) of the 12 probiotic strains showed a concentration of FAAs lower than 800 mg/L (Figure 7). These four strains showed a significantly lower concentration of total FAAs in CKD fecal media compared with the fecal media obtained using HC feces and were, therefore, selected.

### 3.5. Concentration of Short Chain Fatty Acids

**Figure 8:**
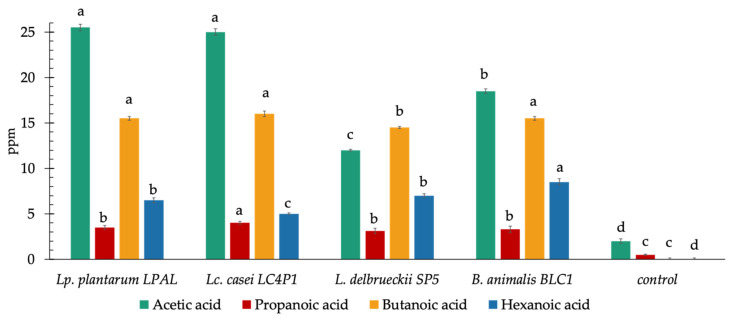
Concentrations of short-chain fatty acids (SCFAs; ppm equivalents) produced by four probiotic strains after 24 h of growth in fecal media obtained from feces of HC patients with the addition of FOS and inulin (2.5 g/L each). A sterilized fecal medium without the addition of bacterial cells was used as the control. Data are reported as the average of one technical triplicate ± SD. ^a–d^ Among same metabolite, different superscript letters showed a significant difference (*p* < 0.05).

Gas chromatography-mass spectrometry (GC-MS) analysis was conducted to evaluate the ability of the four selected probiotic strains to produce short-chain fatty acids (SCFAs). FOS and inulin (each 2.5 g/L) were added to fecal extracts from HC to constitute a HC fecal medium. *B. animalis* BLC1, *L. delbrueckii* SP5, *Lc. casei* LC4P1, and *Lp. plantarum* LPAL were inoculated at a cell density of 7 log CFU/mL. After 24 h at 37 °C, *Lp. plantarum* LPAL and *Lc. casei* LC4P1 were able to produce the highest amounts (ppm equivalents) of acetic acid (Figure 8). Moreover, *Lc. casei* LC4P1 produced higher amounts of both butanoic and propanoic acids than *Lp. plantarum* LPAL. The highest concentration of hexanoic acid was found in the HC fecal media inoculated with *B. animalis* BLC1. Therefore, *B. animalis* BLC1 and *Lc. casei* LC4P1 were selected as the best SCFA-producing probiotics.

### 3.6. Stability Test of the Innovative Synbiotic Formulation

A first innovative synbiotic, named NatuREN P, was constituted as follows:

- *B. animalis* BLC1 (ca. 0.24 g = 10^9^ cells);
- *Lc. casei* LC4P1 (ca. 0.24 g = 10^9^ cells);
- FOS (2.5 g);
- Inulin (2.5 g);
- Pomegranate seed extract (0.2 g);
- Maltodextrins from corn (0.5 g);
- Sodium cyclamate (0.01 g).

As reported above, the initial cell density of the probiotic strains was about 9 log CFU/g of lyophilized cells (minimum daily dose referring to each individual strain). To ascertain the survival of strains in the formulation, their cell density was estimated. The results showed that the cell density of probiotics underwent a decrease of one or more logarithmic cycles after 2 months at both room (20/30 °C) and refrigerated (2/8 °C) temperature.

As Indigo, ResTom, and Bronze are transgenic varieties and their use is, therefore, limited to studies in mouse models, we searched for a mixture of phenolic compounds approaching the profiles of polyphenols and antioxidants of transgenic tomato varieties. Our previous results [27,34] showed that 10% red grape skin extract was able to reduce intestinal inflammation in a colitis mouse model. Based on the polyphenol profiles of Bronze tomato and red grape skin (Supplementary Table S3), we decided to use a polyphenol mixture (named Polimix), simultaneously approaching the polyphenols content of Bronze tomato and red grape skin extract. More specifically, Polimix includes quercetin, resveratrol, and proanthocyanidins in a ratio of 64:23:13. Polimix was added (5 g/L) to artMRS + 2.5 g/L of FOS + 2.5 g/L of inulin, as already performed, to assess the growth of probiotics in the presence of pomegranate seed extract (Pom.S.E.). A control medium without Polimix was used. Acidification and viable cell count of the two best SCFA-producing probiotics (*B. animalis* BLC1 and *Lc. casei* LC4P1) were evaluated after 24 h at 37 °C. The other 17 probiotics reported in Table 1 were only used for comparison. Among bifidobacterial strains, *B. animalis* BLC1 showed the highest ΔpH and a slightly higher cell count than *B. breve* 15A (Supplementary Figure S1A). Among the lactobacilli, *L. delbrueckii* SP5 showed the highest ΔpH (Supplementary Figure S1A), whereas *Lc. casei* LC4P1 had the highest cell count (Supplementary Figure S1B).

Hence, considering the SCFA produced by the 19 probiotics and, in line with the selection criterion previously adopted, a second innovative synbiotic formulation was constituted as follows, and was named NatuREN G:

- *B. animalis* BLC1 (ca. 0.24 g = 10^9^ cells);
- *Lc. casei* LC4P1 (ca. 0.24 g = 10^9^ cells);
- FOS (2.5 g);
- Inulin (2.5 g);
- Quercetin (0.064 g);
- Resveratrol (0.023 g);
- Grapeseed (*Vitis vinifera L.*) powder extract (0.013 g), proanthocyanidins (tit. 95% d.extr.);
- Maltodextrins from corn (0.5 g);
- Sodium cyclamate (0.01 g).

In contrast to NatuREN P, in NatuREN G, the probiotics’ cell density remained approximately stable after two months of storage at both room (20/30 °C) and refrigerated (2/8 °C) temperatures. After 6 months of storage, a slight decrease in cell density was found, but the values of cell density were higher than 8 log CFU/g. The main findings leading to the constitution of NatuREN G are summarized in Table S4.

### 3.7. Effects of NatuREN G on Fecal Microbiota of HC and CKD Subjects

To evaluate the in vitro effect(s) of NatuREN G on CKD, viable bacterial cell counts were determined after adding fresh feces from HC and CKD subjects to fecal media, with the aim of mimicking the composition of gut microbial community. Cultivable bacteria in HC and CKD subjects were estimated at baseline (T0) and after 24 h of incubation at 37 °C, with or without NatuREN G, under anaerobic conditions.

**Figure 9:**
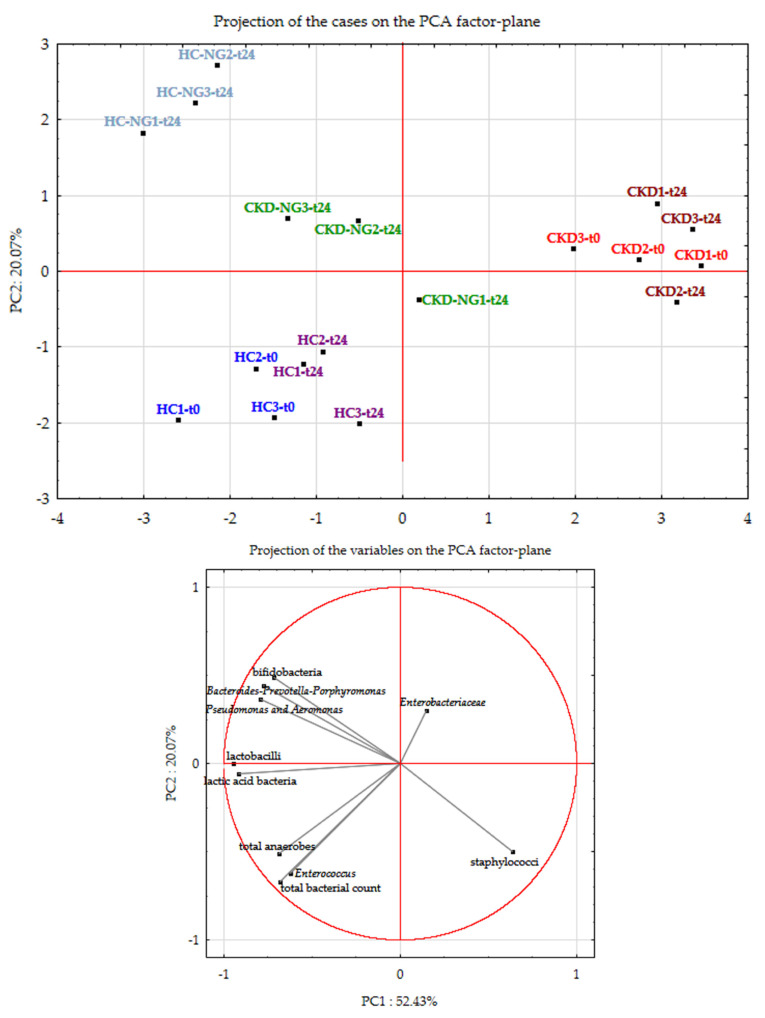
Principal components analysis (PCA) of the main cultivable bacteria found in fecal batches fabricated using sterilized fecal media with the addition of feces from healthy (HC) or nephropathic (CKD) subjects. Data obtained from three independent analyses were collected from baseline samples (sterilized fecal media with fresh feces added, named t0), after 24 h of incubation at 37 °C (t24), and 24 h at 37 °C after adding the innovative synbiotic NatuREN G (NG-t24).

The results of cell densities of the main gut cultivable bacterial groups were elaborated using principal components analysis (PCA). At T0, CKD samples were mainly characterized by high *Enterobacteriaceae* and staphylococci and low lactic acid bacteria (LABs) and lactobacilli (Figure 9). The opposite was found in HC samples at T0. The addition of NatuREN G determined after 24 h showed a shift in CKD samples (CKD-NG-t24) toward the side of the PCA factor-plan wherein all the HC samples had fallen. This was mainly determined by the increase in the viable cell count of both LABs and lactobacilli. Differently, 24 h after adding NatuREN G to HC samples (HC-NG-t24), an increase in viable cell count of the groups *Bacteroides*–*Prevotella*–*Porphyromonas*, *Pseudomonas*–*Aeromonas*, and particularly of the bifidobacterial taxa, was found. This increase was related to decreases in total bacterial, total anaerobes, and *Enterococcus*. Few differences in the HC and CKD samples where NatuREN G had not been added were found compared with T0.

**Figure 10:**
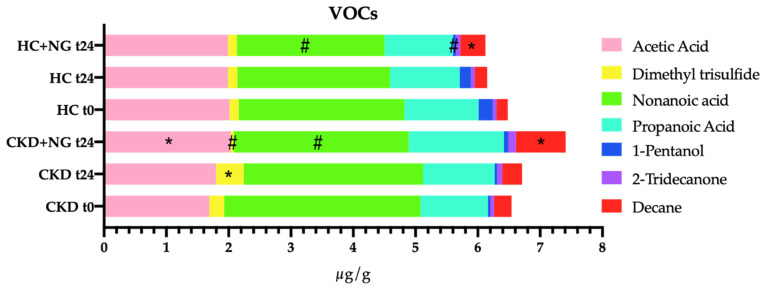
Concentrations of some volatile organic compounds (VOCs; μg/g) found in fecal batches obtained by adding fresh feces from a healthy (HC) or nephropathic (CKD) subject to fecal media obtained with pooled and sterilized HC and CKD fecal extracts. The analysis was conducted immediately on fecal batches (t0) after 24 h without adding the innovative synbiotic NatuREN G (t24), and 24 h after adding the innovative synbiotic NatuREN G (+NGt24). Data are presented as the average of one biological triplicate ± SD. * significantly increased VOC compared with the relative t0; #, significantly decreased VOC compared with the relative t0 (Tukey’s test, *p* < 0.05).

The analysis of volatile organic compounds (VOCs) showed that NatuREN G produced significant variations in VOCs, particularly in fecal batches obtained from CKD patients (CKD + NGt24). Few differences (e.g., decreases in nonanoic acid and 1-pentanol) were found in batches obtained from HC fecal extracts (Figure 10). Decane increased in HC (HC + NGt24) and CKD (CKD + NGt24) fecal batches after 24 h of the addition of the innovative synbiotic. Similar to HC + NG, the nonanoic acid decreased in CKD at t24. Different from HC + NGt24, acetic acid was significantly increased in CKD + NGt24 batches. Interestingly, CKD + NGt24 contained a higher concentration of propanoic acid than HC + NGt24. Additionally, in CKD samples where NatuREN G was not added, an increase in dimethyl trisulfide was found. Conversely, dimethyl trisulfide was significantly decreased after 24 h of incubation of the CKD fecal batch with the addition of NatuREN G.

**Figure 11:**
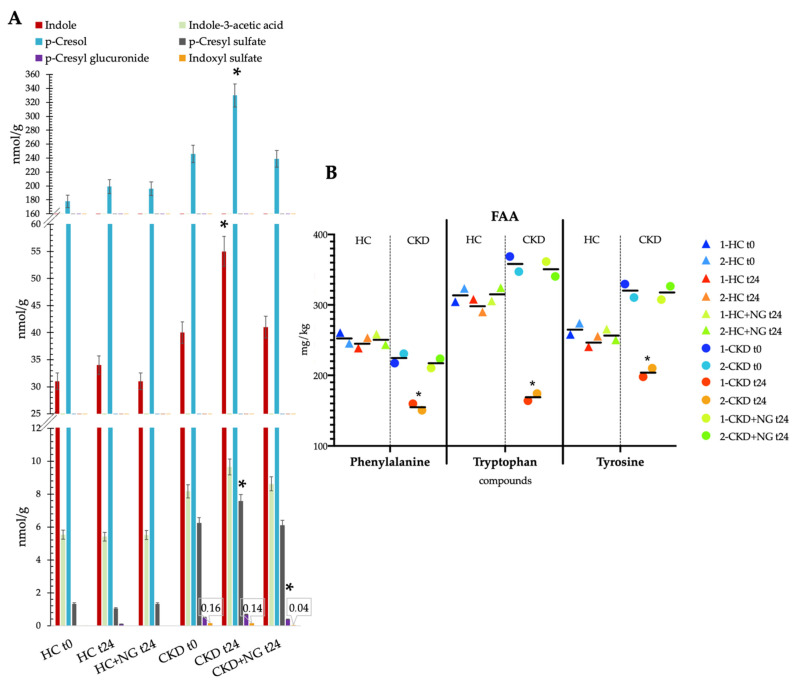
Fecal metabolites (nmol/g wet fecal batch) detection of (**A**) uremic toxins by nuclear magnetic resonance (NMR) and (**B**) uremic toxins precursors by amino acid analysis in chronic kidney disease (CKD) fecal batches before (t0) and after simulated (in vitro) colonic fermentation (t24; 24 h at 37 °C) with and without the innovative synbiotic NatuREN G (NG). Fecal batches with healthy subjects (HC) feces were used as the control. The concentration of NMR compounds is summarized as the average of two independent analyses ± SD. The concentration of FAAs is singularly reported (average expressed as a middle bar). * Significantly different compounds’ concentrations (multiple t-test using the Sidak–Bonferroni correction, *p* < 0.05) compared with the relative baseline (t0).

An analysis of uremic toxins by nuclear magnetic resonance (NMR) was carried out with fresh fecal samples obtained from CKD and HC subjects to evaluate the effects of NatuREN G after 24 h of incubation. Parallel fecal samples, without the addition of NatuREN G, were used as controls. The uremic toxin detection was also evaluated immediately after the addition of fresh feces to fecal media (t0). No significant differences were found in HC samples, comparing concentrations of uremic toxins in both the incubated HC samples (with and without NatuREN G, HCt24 and HC + NGt24, respectively) with the baseline values (HCt0) (Figure 11A). Conversely, indole, *p*-cresol, and *p*-cresyl sulfate significantly increased after 24 h of incubation in CKD samples without the addition of NatuREN G (CKDt24). Furthermore, a significant decrease in indoxyl sulfate was detected comparing CKD + NGt24 to the relative baseline samples (CKDt0) (Figure 11A). The analysis of free amino acids (FAAs) of the above-described fresh fecal samples showed that phenylalanine, tryptophan, and tyrosine significantly decreased in CKD samples with NatuREN G (CKD + NGt24) compared with both CKDt0 and CKDt24 (Figure 11B). On the contrary, no differences were found comparing all the HC samples.

## 4. Discussion

To develop an innovative synbiotic with the prospective purpose of being used in nephropathy, we evaluated different probiotics, antioxidants, and prebiotics. Starting from the evidence that CKD patients show an increased incidence of oxidative stress [35] and that, in nephropathy, oxidative unbalance is due to increased production of reactive oxygen species [36], we characterized different antioxidants for the development of an innovative synbiotic. The applied selection methods are primarily based on the evaluation of probiotics’ ability to grow in the presence of antioxidants and prebiotics. Some antioxidants have antimicrobial activities [37,38,39] and, for this reason, the first analyses were carried out on probiotics in the presence of antioxidants. Twenty-five probiotics, previously isolated from animal and human feces or commercially available formulations, were used. The tested bacteria belonged to the two main groups of probiotics, *Bifidobacteria* and lactobacilli. In the two last decades, evidence widely highlighted the beneficial roles of these two taxa on host health [40,41,42]. Both of these probiotics are recognized as the main saccharolytic bacteria of a healthy human gut [43]. We specifically focused on them also because of the critical lack of *Bifidobacterium* and lactobacilli frequently found in CKD [8]. We selected those probiotics that, in the presence of antioxidants, showed the highest degree of acidification. Then, the same criterion was adopted for prebiotics evaluation, with the aim of selecting high SCFA-producing bacteria. In healthy conditions, SCFAs are the main energy source for intestinal epithelial cells; additionally, they are primarily involved in maintaining intestinal pH within optimal ranges [18].

Subsequently, we evaluated the in vitro effect(s) of the resulting combination of antioxidants and prebiotics. Experimental treatments were initially tested in fecal media prepared from feces collected from healthy subjects. Subsequently, the same was also performed with fecal media prepared from feces collected from CKD patients at stage IIIb-IV, i.e., the worst condition before the ESRD stage when dialysis becomes essential. In the evaluation of the obtained results, we underline the widely recognized limitations that are innate in our experimental conditions due to the impact of sampling fecal microbiota communities [44]. However, many (12 out of 25) of the evaluated probiotics showed promising results, at least in our in vitro analyses. Therefore, the total free amino acids (FAAs) analysis was an intermediate step, playing a pivotal role in decreasing the mainly proteolytic metabolism typical of nephropathy [45]. In CKD, the dysbiotic, highly proteolytic, microbial patterns determine the increase in uremic toxins, e.g., IS and pCS, produced from urea and other nitrogenous compounds [8]. Moreover, due to low kidney functionality, CKD patients are unable to excrete waste metabolites (including urea, ammonia, and uremic toxins), which, in turn, contribute to pH increase in the intestinal environment [7]. Additionally, urea and ammonia are primarily involved in the tight junctions’ disruption in CKD [46]. Based on these considerations, the FAAs analysis allowed us to reduce the number of selected probiotics from 12 to 4 strains. In fecal media obtained using CKD feces, only *B. animalis* BLC1, *L. delbrueckii* SP5, *Lc. casei* LC4P1, and *Lp. plantarum* LPAL showed lower proteolytic activity. This result confirmed the evidence of the high protease/peptidase activity of lactic acid bacteria. The proteolytic activity of lactic acid bacteria plays a role in cheese ripening [47] and, through hydrolysis of gliadins and related epitopes, in ameliorating the symptomatology related to gluten malabsorption and celiac disease [48,49].

Therefore, the last step in the adopted workflow was the characterization of the ability of the four selected probiotics to produce SCFA, given the evidence discussed above [17,20,21]. The largest spectrum of SCFAs was achieved using *B. animalis* BLC1 and *Lc. casei* LC4P1. Thus, a first innovative synbiotic was developed (NatuREN P), which comprised *B. animalis* BLC1, *Lc. casei* LC4P1, FOS, inulin, and pomegranate seed extract. The stability tests showed that, unfortunately, a critical decrease in the probiotics’ viability occurred after two months of storage. For this reason, based on the promising results obtained upon administration of polyphenols-enriched tomato and red grape skin extracts in colitis mouse models [27,34], we then evaluated a polyphenolic mixture, named Polimix, including quercetin, resveratrol, and proanthocyanidins. Polimix simulates the polyphenol profile classes of transgenic tomato varieties and red grape skin. Thus, a second innovative synbiotic was developed and named NatuREN G, which comprised *B. animalis* BLC1, *Lc. casei* LC4P1, FOS, inulin, quercetin, resveratrol, proanthocyanidins from grapeseed (*Vitis vinifera* L.) powder extract, maltodextrins from corn, and sodium cyclamate. The stability tests performed on NatuREN G showed that the probiotic cell density remained approximatively stable for two months of product storage, at both room and refrigerated temperatures. Additionally, only a slight decrease in cell density was found after six months of storage.

Hence, the development of the present synbiotic occurred within an ongoing research project in which different treatments were being administered to CKD patients. The common aim of previous works was to evaluate whether different dietary interventions with probiotics and prebiotics might ameliorate pathognomonic features in nephropathy. The literature in this field reports conflicting results, where not all treatments reached the expected outcomes. In line with this, Takayama et al. found that the administration of *Bifidobacterium longum* was effective in decreasing indoxyl sulfate (IS) in hemodialyzed patients [22]. The collected data showed that five weeks of treatment were sufficient to decrease serum levels of IS. Similarly, a mixture of different probiotics, including *Streptococcus*, *Lactobacillus*, and *Bifidobacterium* strains, was administered to hemodialyzed patients [23], even if, in this work, no changes in TMAO levels were found in plasma. Concerning prebiotics, four weeks of treatment with resistant starch showed significant decreases in both the IS and IL-6 plasma levels of hemodialyzed patients [50], while oligofructose-enriched inulin showed its impact by decreasing *p*CS levels [51]. Other trials focused on the effects of FOS administration to CKD patients. In one of these studies, FOS produced a reduction in circulating *p*CS, even if no changes in IS levels were recorded [52]. Conversely, another study using FOS reported an amelioration in inflammatory parameters and endothelial integrity, whereas only slight but not significant differences in *p*CS were found [53].

Before industrial scale-up, NatuREN G was tested in fecal batches prepared from HC and CKD subjects, showing promising results, although confirmation in vivo remains necessary. In detail, a significant increase in both lactic acid bacteria and lactobacilli was found in CKD fecal batches. In addition, in both batches (from HC and CKD) an increase in bifidobacterial species was found after 24 h of the addition of NatuREN G. As these results only partially reproduce the nephropathic intestinal environment, it seems that NatuREN G may be able to modify the microbial balance toward a eubiotic microbiota composition. Notably, the VOCs analysis showed that the innovative synbiotic NatuREN G produced an increase in SCFAs (acetic and propanoic acids) in CKD samples, 24 h after its addition. Additionally, a clear reduction in the uremic toxin concentration was found through NRM analysis and confirmed by amino acids detection. This result suggests that the innovative synbiotic NatuREN G seems to be able to reduce the metabolic pathways leading to uremic toxin precursors, at least in in vitro analyses.

We are conscious that the observed findings need to be confirmed with an in vivo application due to the limitations that markedly characterize in vitro approaches. However, previous trials reported promising outcomes of treatments with synbiotics [24,25,26], possibly due to the synergistic interaction between probiotics and prebiotics. Treatment based on the administration of lactobacilli and bifidobacterial strains together with galacto-oligosaccharides (GOS) decreased *p*CS levels [24]. A similar outcome was obtained in CKD patients (stage IIIb-IV), administering a mixture of *Bifidobacteria* and lactic acid bacteria (lactobacilli and streptococci) with prebiotics (FOS, inulin, and resistant starch) for four weeks [25]. The same was also found in another set of CKD patients, in which a similar synbiotic containing a probiotic consortium (*Bifidobacteria*, lactobacilli, and streptococci) and prebiotics (inulin, FOS, and GOS) produced a reduction in *p*CS [26].

In our opinion, the approach adopted to constitute this innovative synbiotic, following a step-by-step workflow before proceeding with in vivo evaluation, can be considered among the strengths of this study. The probiotics were selected after evaluating their interaction with other constituents and, subsequently, were selected for their biological activities. Moreover, the in vitro evaluation of fecal microbiota allowed us to obtain preliminary evidence about their effects when in the presence of viable microbiota. Nonetheless, some limitations need to be acknowledged. Firstly, although feces were collected in sterile stool containers filled to over four-fifths with the aim of reducing the headspace and processed within 6 h from the collection, the used fecal microbiota could have been different from real intestinal microbiota. Additionally, the in vitro analyses were unable to reproduce all variables characterizing the host and, therefore, the in vivo evaluation of NatuREN G remains essential.

## 5. Conclusions

This study provides evidence that the innovative synbiotic NatuREN G may be able to decrease the level of uremic toxins, at least under in vitro conditions simulating dysbiosis and some of the pathognomonic characteristics linked to nephropathy. The innovative synbiotic produced some shifts in the main microbial groups, favoring saccharolytic ones and suppressing the overgrowth of proteolytic bacteria. This shift might be able to increase the metabolism of SCFA, also leading to restored intestinal pH values, reducing the epithelial layer disruption, and, therefore, delaying ESRD onset and the need for dialysis. However, due to a critical lack of systems reproducing real host physiology, particularly given the biases related to short-term air exposure of collected feces, possibly affecting the composition of fecal microbiota, in vivo evaluation of NatuREN G is essential.

## Figures and Tables

**Table 1:** Acidification of probiotics in artisanal MRS (artMRS) used alone (as a control) or supplemented with pomegranate seed extract (Pom.S.E., 5 g/L), or Pom.S.E. + fructo-oligosaccharides (FOS) (each 5 g/L), or Pom.S.E. (5 g/L) + FOS (2.5 g/L) + inulin (2.5 g/L). The difference in acidification degree (Diff.Ac.Deg.) between the control and the thesis reporting the lowest pH values (Pom.S.E. + FOS+ inulin) is reported in the last column. Data are presented as the average of one biological triplicate ± SD.

Probiotics	pH artMRS *	pH artMRS + Pom.S.E.	pH artMRS + Pom.S.E. + FOS	pH artMRS + Pom.S.E. + FOS + inu	(Diff.Ac.Deg.) pH artMRS − pH artMRS + Pom.S.E. + FOS + inu
*B. animalis 13A*	5.66 ± 0.12 ^a^	5.51 ± 0.11 ^ab^	5.37 ± 0.11 ^bc^	5.26 ± 0.04 ^c^	0.40 ± 0.08
*B. animalis BLC1*	5.31 ± 0.10 ^a^	5.23 ± 0.06 ^a^	5.02 ± 0.11 ^b^	4.86 ± 0.08 ^b^	0.45 ± 0.09
*B. breve 15A*	5.24 ± 0.07 ^a^	5.15 ± 0.10 ^a^	4.91 ± 0.12 ^bc^	4.77 ± 0.03 ^c^	0.47 ± 0.05
*L. acidophilus LA3*	4.83 ± 0.09 ^a^	4.78 ± 0.10 ^ab^	4.65 ± 0.04 ^b^	4.59 ± 0.11 ^b^	0.24 ± 0.10
*L. delbrueckii SP5*	5.34 ± 0.11 ^a^	5.21 ± 0.03 ^a^	5.02 ± 0.12 ^b^	4.86 ± 0.09 ^b^	0.48 ± 0.10
*Lc. casei BGP93*	4.92 ± 0.06 ^a^	4.94 ± 0.08 ^a^	4.81 ± 0.11 ^ab^	4.74 ± 0.07 ^b^	0.18 ± 0.07
*Lc. casei LC4P1*	4.93 ± 0.08 ^a^	4.95 ± 0.06 ^a^	4.70 ± 0.11 ^b^	4.56 ± 0.05 ^b^	0.37 ± 0.07
*Lc. paracasei 14A*	5.09 ± 0.05 ^a^	4.92 ± 0.05 ^b^	4.80 ± 0.04 ^c^	4.69 ± 0.11 ^c^	0.40 ± 0.08
*Lc. rhamnosus LRB*	4.91 ± 0.10 ^a^	4.99 ± 0.08 ^a^	4.88 ± 0.08 ^ab^	4.81 ± 0.04 ^b^	0.10 ± 0.07
*Lc. rhamnosus SP1*	4.95 ± 0.10 ^a^	4.98 ± 0.02 ^a^	4.88 ± 0.11 ^ab^	4.80 ± 0.11 ^b^	0.15 ± 0.11
*Lp. plantarum 12A*	5.10 ± 0.10 ^a^	4.92 ± 0.02 ^b^	4.81 ± 0.03 ^c^	4.69 ± 0.12 ^c^	0.41 ± 0.11
*Lp. plantarum 3ON*	5.03 ± 0.08 ^a^	4.95 ± 0.10 ^ab^	4.84 ± 0.05 ^bc^	4.69 ± 0.10 ^c^	0.34 ± 0.09
*Lp. plantarum 4.1*	5.42 ± 0.10 ^a^	5.37 ± 0.10 ^ab^	5.26 ± 0.06 ^bc^	5.19 ± 0.04 ^c^	0.23 ± 0.07
*Lp. plantarum LPAL*	4.96 ± 0.07 ^a^	4.91 ± 0.10 ^ab^	4.78 ± 0.06 ^b^	4.66 ± 0.02 ^c^	0.30 ± 0.05
*Lp. plantarum ONI17*	4.98 ± 0.11 ^a^	4.97 ± 0.07 ^a^	4.90 ± 0.11 ^ab^	4.83 ± 0.05 ^b^	0.15 ± 0.08
*Lp. plantarum ONI3*	5.01 ± 0.06 ^a^	4.91 ± 0.05 ^ab^	4.78 ± 0.08 ^bc^	4.67 ± 0.03 ^c^	0.34 ± 0.05
*Lp. plantarum VEGI1*	4.97 ± 0.12 ^a^	4.95 ± 0.02 ^a^	4.78 ± 0.10 ^ab^	4.67 ± 0.11 ^b^	0.30 ± 0.12
*Lp. plantarum VEGII1*	4.99 ± 0.05 ^a^	5.02 ± 0.11 ^ab^	4.89 ± 0.04 ^b^	4.83 ± 0.12 ^b^	0.16 ± 0.09
Synbio 100	4.93 ± 0.10 ^a^	5.06 ± 0.10 ^a^	4.34 ± 0.04 ^b^	4.10 ± 0.10 ^c^	0.83 ± 0.10

* artMRS = artisanal MRS (2 g/L of glucose). ^a–c^ Values within the same column showing different superscript letter are significantly different (Tukey’s test, *p* < 0.05). Within the last column, underlined values showed the selected probiotics.

## Data Availability

The data presented in this study are available on request from the corresponding author.

## Supplementary Materials

The following are available online at https://www.mdpi.com/article/10.3390/microorganisms9061316/s1. Table S1: Main polyphenol classes detected in wild type (WT) tomato (cv. Moneymaker) and the high polyphenol enriched transgenic lines Indigo, ResTom, and Bronze. Table S2: Analysis of pomegranate juice extracted from different varieties, specifically Kamel, Emek, Ako, and Wonderful one. Table S3: Comparison between the high-polyphenol-enriched Bronze line and grape (white and red) skin in terms of the main classes of polyphenols. Table S4: Main findings leading to the constitution of NatuREN G. Figure S1: (A) Change in pH (ΔpH) after 24 h of probiotics growth at 37° C in artisanal MRS ((artMRS), 2 g/L of glucose) with the addition of a polyphenolic mixture (5 g/L; quercetin, resveratrol, and proanthocyanidins; ratio 64:23:13, respectively). (B) Probiotic cell density (log CFU/mL) after 24 h of growth in artMRS (2 g/L of glucose) used alone or supplemented with the polyphenolic mixture (5 g/L; quercetin, resveratrol, and proanthocyanidins; ratio 64:23:13, respectively).

## Appendix A

### Appendix A.1. Pomegranate Juice Extraction

All pomegranate fruits were collected during the 2015–2016 commercial harvest from Cairo & Doutcher farm in Copertino (Lecce, Italy). Freshly harvested fruits of the same size, without any physical defects, were randomly selected, washed with tap water, dried, cut in half, and squeezed by a professional electric juicer (Fimar, Villa Verucchio (Rimini), Italy). The juice was immediately stored at −80 °C until analysis. Four independent extractions (biological replicas) were performed using ten fruits each. All analyses were performed in triplicate (sampling replicas) for each extract.

### Appendix A.2. Determination of Moisture, Ash, Total Soluble Solids, and pH

Moisture was determined gravimetrically after drying 1.0 g aliquots of the pomegranate extract at 105 °C in a Büchi TO-50 infrared dryer (BÜCHI Labortechnik AG, Flawil, Switzerland). Total soluble solids were measured using a digital refractometer (DBR95 Giorgio Bormac S.r.l., Carpi (Modena), Italy) and the results are expressed as °Brix. Ash content was determined on 5.0 g aliquots of each sample using a muffle furnace at 525 °C for 6 h, according to the AOAC (2005) method [54]. The ash content is expressed as g per 100 g of fresh weight (g/100 g f.w.). The pH of the extract was determined at room temperature using a pH meter (Mettler Toledo, Columbus, OH, USA).

### Appendix A.3. Total Carbohydrates, Total Polysaccharides, Pectins, and Starch Determination

Total carbohydrates were determined with the phenol-sulfuric acid method using a Beckman DU 650 spectrophotometer (Beckman Coulter Inc., Brea, CA, USA) according to Nielsen [55]. Total polysaccharide content was measured with the same method on precipitates obtained by mixing 5 mL juice aliquots with ethanol (70% final concentration) and centrifuging at 6000× *g* for 10 min. Pectins were quantified using the spectrophotometric method proposed by Blumenkrantz and Asboe-Hansen [56]. Total starch was determined using the Megazyme Total Starch Assay Kit (Megazyme International Ireland, Bray, Ireland), based on the AOAC official methods 996.11 [57].

### Appendix A.4. Analysis of Soluble Sugars and Organic Acids

Soluble sugars and organic acids in the extracts were analyzed using high-performance liquid chromatography (HPLC), as described by Castellari et al. [58]. Identification and quantification of sugars and organic acids were performed by comparison of peak retention times and areas with those of external standards.

### Appendix A.5. Ascorbic Acid (AsA) and Dehydroascorbic Acid (DHA) Determination

AsA and DHA were extracted using 6% metaphosphoric acid. Their concentrations were determined on triplicate aliquots of the extract (0.1 g) using a Beckman DU 650 spectrophotometer, set at a wavelength of 525 nm [59].

### Appendix A.6. Extraction and Determination of Soluble and Insoluble-Bound Phenolics

Phenols were extracted according to Adom et al. [60]. Briefly, 0.05 g of extracted juice was mixed with 1 mL of 80% (*v*/*v*) ethanol and extracted at room temperature for 10 min under constant shaking (300 rpm). After centrifugation (2500× *g* for 10 min), the supernatant containing the soluble phenolic compounds was recovered. The extraction was repeated twice, and the supernatants were combined.

The insoluble-bound phenols were extracted from the pellets resulting from the soluble phenols extraction. The pellets were sequentially washed twice with 100% methanol and a mixture of chloroform/methanol/water (1:1:1, *v*/*v*) and once with 100% acetone. After each washing step, the extracts were centrifuged (8800× *g* for 7 min) and the supernatants discarded. The residues were treated with 1.0 M NaOH (1 mL) at room temperature for 1 h under shaking and a nitrogen atmosphere. The mixtures were acidified to pH 2 (12 M HCl) and extracted three times with 500 µL of 100% ethyl acetate. The ethyl acetate fraction was evaporated to dryness. Phenolic compounds in dried extract were dissolved in 500 µL of 80% ethanol (*v*/*v*).

Phenolic content was determined on each extract (soluble and insoluble-bound phenolics) according to the method of Xu et al. [61]. Briefly, 50 µL of extract was mixed with 50 µL of Folin–Ciocalteu’s phenol reagent and 450 µL distilled water. The mixture was kept at room temperature for 5 min, and then 500 µL of sodium carbonate (7% ×) was added. After brining the mixture to final volume (1250 µL) with distilled water, the mixture was left at room temperature in the dark for 90 min. The absorbance was read at 750 nm in a Beckman DU650 spectrophotometer. The amounts of total phenols were calculated using gallic acid (GA) as the calibration standard within the range of 0–12 µg GA/100 µL. The results are expressed as mg GA equivalents (GAE)/g fresh weight (f.w.).

### Appendix A.7. Extraction and Determination of Flavonoids and Proanthocyanidins

We mixed 0.3 g of each juice with 1.5 mL of 100% methanol (*v*/*v*). The mixture was then shaken at 4 °C for 16 h, then centrifuged at 8800× *g* for 10 min. All supernatants were recovered and used for determining total flavonoid and proanthocyanidin contents.

The total flavonoid content was determined as described by Zhishen et al. [62]. We diluted 50 µL of extract with distilled water to a final volume of 500 µL, and 30 µL of 5% NaNO_2_ was added. After 5 min, 60 µL of 10% AlCl_3_ was added, followed after a further 6 min with the addition of 200 µL of 1M NaOH and 210 µL of distilled water. The absorbance was read at 510 nm in a Beckman DU650 spectrophotometer. The linear calibration curve was constructed using catechin (0–400 µg/mL). The results are expressed as mg of catechin equivalents (CE)/g f.w. Condensed tannins were determined according to Broadhurst and Jones [63]. We mixed 100 µL of extract, contained in a test tube covered with aluminum foil, with 600 µL of 4% vanillin–methanol solution and then with 300 µL of 12 M hydrochloric acid. The mixture was allowed to stand for 15 min at 20 °C in the dark. The absorbance of the mixture was read at 500 nm in a Beckman DU650 spectrophotometer. The results are expressed as mg of catechin equivalents (CE)/g f.w., calculated based on a calibration curve (from 3.9 to 250 µg catechin/mL).

### Appendix A.8. Extraction and Determination of Total Anthocyanins

Total anthocyanins were extracted according to Zhao et al. [64]. Briefly, 0.5 g of each juice was mixed with 15 mL of methanol containing 0.1% HCl. The samples were stirred at room temperature and in the dark for 30 min. Subsequently, they were centrifuged at 3900× *g* for 30 min at 4 °C in a Beckman AllegraTM X-22 centrifuge. Supernatants were dried by evaporation at a constant temperature of 35 °C. The dried residue was suspended in 1 mL of distilled water.

The total anthocyanins (TA) were estimated using the pH differential method with two buffer systems: potassium chloride (25 mM) buffer pH 1.0 and sodium acetate (0.4 M) buffer pH 4.5 [65]. Briefly, 0.1 mL aliquots of each fraction were mixed with 0.9 mL corresponding buffers. The samples were left at room temperature and in the dark for 15 min and their absorbance values at 510 and 700 nm was determined (reading against water). The absorbance values were calculated as follows:

A = (A510 nm − A700 nm) pH 1.0 − (A510 nm − A700 nm) pH 4.5

For the determination of total anthocyanins content, the following formula was used:

TA (mg/L) = [A × MW × DF × 1000] × 1/MA

where A is absorbance, MW is the molecular weight of cyanidin-3-glucoside (449.2 g/mol), DF is the dilution factor, and MA is the molar absorptivity coefficient of cyanidin-3-glucoside (26,900).

The final results are expressed as µg of cyanidin-3-glucoside/g f.w.

### Appendix A.9. Antioxidant Activity Determination

Molecules with antioxidants activity were extracted according to Hdider et al. [66], with slight modifications. Briefly, 0.4 g of each juice was sequentially extracted with 100% methanol and 100% acetone (liposoluble antioxidants) under constant shaking (300 rpm) at room temperature in the dark for 1 h. Samples were centrifuged at 2500× *g* for 10 min. Supernatants were recovered and used for the antioxidant activity assay. Hydrophilic antioxidant activity (HAA) and lipophilic antioxidant activity (LAA) were measured by the Trolox equivalent antioxidant capacity (TEAC) assay as described by Re et al. [67], using the ABTS discoloration method. The absorbance decrease was measured at 734 nm in a Beckman DU650 spectrophotometer. The linear calibration curves ranged from 0 to 15 µM Trolox. The antioxidant activity of the samples was calculated based on the inhibition exerted by standard Trolox concentrations at 734 nm, with inhibition time fixed at 10 min. Total antioxidant activity (TAA) was calculated as the sum of HAA and LAA. Results are expressed as µmol of Trolox equivalents (TE)/g f.w.
