# Supplementary material for: In Vitro Selection of Probiotics, Prebiotics, and Antioxidants to Develop an Innovative Synbiotic (NatuREN G) and Testing Its Effect in Reducing Uremic Toxins in Fecal Batches from CKD Patients
Source: Microorganisms. 2021 Jun 17;9(6):1316. doi: 10.3390/microorganisms9061316 (PMC8235484; doi:10.3390/microorganisms9061316)
Supplement: Supplementary file 1 [file microorganisms-09-01316-s001.zip › Supplementary_Table_S1.pdf]

Supplementary Table S1. Main polyphenol classes detected in wild type tomato (WT; cv. Moneymaker) and the high polyphenol enriched transgenic lines Indigo, ResTom, and Bronze.

Compounds (mg/g DW)	WT	Indigo	ResTom	Bronze
Flavonoids	0.46 ± 0.029^{c}	20.39 ± 3.35^{a}	2.40 ± 0.23^{c}	14.52 ± 1.12^{b}
Anthocyanins	0^{c}	4.94 ± 0.077^{a}	0^{c}	2.5 ± 0.04^{b}
Stilbenoids	0^{c}	0^{c}	9.12 ± 0.78^{a}	5.49 ± 1.49^{b}

Values within the same row showing different superscript letter are significantly different (Tukey test, $p < 0.05$).
