# Supplementary material for: In Vitro Selection of Probiotics, Prebiotics, and Antioxidants to Develop an Innovative Synbiotic (NatuREN G) and Testing Its Effect in Reducing Uremic Toxins in Fecal Batches from CKD Patients
Source: Microorganisms. 2021 Jun 17;9(6):1316. doi: 10.3390/microorganisms9061316 (PMC8235484; doi:10.3390/microorganisms9061316)
Supplement: Supplementary file 1 [file microorganisms-09-01316-s001.zip › Supplementary_Table_S2.pdf]

Supplementary Table S2. Analysis of pomegranate juice extracted from different varieties, specifically Kamel, Emek, Ako, and Wonderful one. Values represent mean ± S.E. of four independent biological replicates. Values within the same row showing different superscript letter are significantly different (Tukey test, $p < 0.05$). GAE, Gallic acid equivalents; CE, Catechin Equivalents; C3GE, cyanidin-3-glucoside equivalents; TE, Trolox equivalents; nd, not detected; fw, fresh weight.

**Component**	**Kamel**	**Emek**	**Ako**	**Wonderful One**
Moisture (%)	86.3 ± 0.4 ^{a}	87.0 ± 0.5 ^{a}	86.5 ± 0.5 ^{a}	86.9 ± 0.5 ^{a}
Ash (%)	0.6 ± 0.1 ^{a}	0.5 ± 0.1 ^{a}	0.6 ± 0.1 $^{a16.3}$	0.6 ± 0.1 ^{a}
Total Soluble Solids (°Brix)	16.3 ± 0.3 ^{a}	16.0 ± 0.3 ^{a}	15.9 ± 0.3 ^{a}	16.3 ± 0.4 ^{a}
pH	3.6 ± 0.2 ^{ab}	4.1 ± 0.3 ^{a}	4.1 ± 0.2 ^{a}	3.5 ± 0.3 ^{b}
Total carbohydrates (mg (g^{-1}) fw)	138.1 ± 11.2 ^{a}	141.9 ± 3.9 ^{a}	114.4 ± 3.4 ^{b}	149.1 ± 15.3 ^{a}
Soluble sugars (mg (g^{-1}) fw)				
Glucose	46.2 ± 1.6 ^{b}	50.3 ± 1.3 ^{a}	40.9 ± 1.9 ^{c}	41.4 ± 1.0 ^{c}
Fructose	54.6 ± 1.9 ^{a}	57.3 ± 2.5 ^{a}	47.8 ± 1.0 ^{b}	47.6 ± 1.1 ^{b}
Sucrose	traces	traces	traces	traces
Total polysaccharides (mg (g^{-1}) fw)	33.2 ± 4.5 ^{a}	38.8 ± 4.1 ^{a}	33.9 ± 4.2 ^{a}	32.8 ± 3.2 ^{a}
Pectins	14.9 ± 1.7 ^{a}	15.2 ± 1.7 ^{a}	15.5 ± 1.3 ^{a}	15.4 ± 1.2 ^{a}
Starch	nd	nd	nd	nd
Organic acids (mg (g^{-1}) fw)				
Citric acid	20.4 ± 1.1 ^{a}	6.2 ± 0.8 ^{c}	6.3 ± 0.9 ^{c}	19.3 ± 1.6 ^{b}
Succinic acid	6.0 ± 0.5 ^{a}	5.5 ± 0.6 ^{a}	5.7 ± 0.4 ^{a}	6.9 ± 1.3 ^{a}
Malic acid	2.4 ± 0.3 ^{b}	3.9 ± 0.3 ^{a}	4.3 ± 0.6 ^{a}	2.5 ± 0.3 ^{b}
Tartaric acid	traces	traces	traces	traces
Vitamin C (mg(g^{-1}) fw)				
Ascorbic acid	0.42 ± 0.02 ^{d}	0.58 ± 0.02 ^{c}	0.95 ± 0.03 ^{a}	0.71 ± 0.03 ^{b}
Dehydroascorbic acid	0.20 ± 0.03 ^{b}	0.70 ± 0.01 ^{a}	0.23 ± 0.02 ^{b}	0.06 ± 0.01 ^{c}
Phenolics (mg GAE(g^{-1}) fw)				
Soluble	4.6 ± 0.7 ^{b}	9.7 ± 0.6 ^{a}	9.6 ± 0.3 ^{a}	9.2 ± 0.6 ^{a}
Insoluble	0.13 ± 0.01 ^{a}	0.09 ± 0.01 ^{b}	0.07 ± 0.01 ^{b}	0.13 ± 0.01 ^{a}
Total flavonoids (mg CE(g^{-1}) fw)	0.9 ± 0.1 ^{b}	1.4 ± 0.1 ^{a}	0.9 ± 0.1 ^{b}	1.1 ± 0.3 ^{ab}
Condensed tannins (mg CE (g^{-1}) fw)	0.6 ± 0.1 ^{c}	1.5 ± 0.1 ^{a}	1.0 ± 0.1 ^{b}	1.2 ± 0.2 ^{b}
Total anthocyanins (mg C3GE(g^{-1}) fw)	0.44 ± 0.03 ^{b}	0.26 ± 0.03 ^{c}	0.22 ± 0.01 ^{c}	0.61 ± 0.09 ^{a}
Antioxidant activity (μmol TE (g^{-1}) fw)				
Hydrophilic	77 ± 8 ^{a}	68 ± 4 ^{a}	66 ± 6 ^{a}	66 ± 11 ^{a}
Lipophilic	1.4 ± 0.3 ^{a}	0.7 ± 0.1 ^{b}	0.4 ± 0.1 ^{b}	0.9 ± 0.4 ^{ab}
