# Supplementary material for: In Vitro Selection of Probiotics, Prebiotics, and Antioxidants to Develop an Innovative Synbiotic (NatuREN G) and Testing Its Effect in Reducing Uremic Toxins in Fecal Batches from CKD Patients
Source: Microorganisms. 2021 Jun 17;9(6):1316. doi: 10.3390/microorganisms9061316 (PMC8235484; doi:10.3390/microorganisms9061316)
Supplement: Supplementary file 1 [file microorganisms-09-01316-s001.zip › Supplementary_Table_S3.pdf]

Supplementary Table S3. Comparison between the high polyphenol enriched Bronze line and grape (white and red) skin in term of main classes of polyphenols classes.

Compounds (mg/g DW)	Bronze	Red Grape	White Grape
Flavonoids	14.52 ± 1.12	1.32 ± 0.92	0.60 ± 0.26
Anthocyanins	2.5 ± 0.04	5.09 ± 0.4	0
Stilbenoids	5.49 ± 1.49	0.24 ± 0.044	0.11 ± 0.016
