# Supplementary material for: In Vitro Selection of Probiotics, Prebiotics, and Antioxidants to Develop an Innovative Synbiotic (NatuREN G) and Testing Its Effect in Reducing Uremic Toxins in Fecal Batches from CKD Patients
Source: Microorganisms. 2021 Jun 17;9(6):1316. doi: 10.3390/microorganisms9061316 (PMC8235484; doi:10.3390/microorganisms9061316)
Supplement: Supplementary file 1 [file microorganisms-09-01316-s001.zip › Supplementary_Table_S4.pdf]

Supplementary Table S4. Main findings leading to constitution of NatuREN G.

Deliverable	Description	Outcome	Notes
Selection of antioxidants	Selection of tomato extracts from wild type, Indigo, ResTom, and Bronze varieties<br>Pomegranate juice of Kamel, Emek, Ako, and Wonderful one	Selected antioxidant matrices for further analysis: Indigo, ResTom, and Bronze (among tomato) and Kamel and Wonderful one (among pomegranate).<br><br>Reducing from 25 to 19 probiotic strains.	Excluded strains:<br>- *L. acidophilus ATCC4356*<br>- *Lc. casei ATCC393*<br>- *Lc. rhamnosus ATCC7469*<br>- *Lp. plantarum 8VEG3C*<br>- *Ls. reuteri ATCC23272*<br>- *Ls. reuteri TMW*
Selection of prebiotics	FOS, inulin, and β-glucans	Among prebiotics, β-glucans were excluded.	
Evaluation of combined antioxidants with prebiotics	Pom.S.E.* + FOS (with and without inulin)	Best thesis: Pom. S. E. + FOS + inulin.<br><br>Reducing from 19 to 12 probiotic strains.	Excluded strains:<br>- *L. acidophilus LA3*<br>- *Lc. casei BGP93*<br>- *Lc. rhamnosus LRB*<br>- *Lc. rhamnosus SP1*<br>- *Lp. plantarum 4.1*<br>- *Lp. plantarum ONI17*<br>- *Lp. plantarum VEGII1*
Evaluation of probiotic growth in HC FM[§]	•HC FM+ FOS+ inulin (**The-1**)<br>•HC FM + FOS+ inulin+ Pom.S.E. (**The-2**)<br>•HC FM + FOS+ inulin+ ResTom (**The-3a**)<br>•HC FM + FOS+ inulin+ Indigo (**The-3b**)<br>•HC FM + FOS+ inulin+ Bronze (**The-3c**)<br>•HC FM + FOS+ inulin+ WT tomato (**The-3d**)	Best theses: The-1 and The-2.<br><br>No probiotic strain was excluded.	
Evaluation of probiotic growth in CKD FM[$]	•FM(CKD)+ FOS+ inulin (**The-1**)<br>•FM(CKD) + FOS+ inulin+ Pom.S.E. (**The-2**)	Except for one strain (*B. animalis 13A*), all the probiotics reported an increase of at least one logarithmic cycle in The-2.	Considering the goal of including antioxidant(s) in the innovative synbiotic formulation, the thesis The-2 was used to proceed with further analyses.
Evaluation of probiotics in terms of release of free amino acids	The analysis was performed comparing CKD with HC FM.	Reducing from 12 to 4 probiotic strains.	Excluded strains:<br>- *B. animalis 13A*<br>- *B. breve 15A*<br>- *Lc. paracasei 14A*<br>- *Lp. plantarum 12A*<br>- *Lp. plantarum 3ON*<br>- *Lp. plantarum ONI3*<br>- *Lp. plantarum VEGI1*<br>- *Synbio 100*

Evaluation of probiotics in terms of production of SCFA	GC-MS£ analysis was carried out on HC FM added with FOS and inulin (each 2.5 g/L) and singly inoculated with 4 selected probiotics.	Highest acetic acid producers: *Lp. plantarum* LPAL and *Lc. casei* LC4P1.<br>*Lc. casei* LC4P1 produced higher amount of both butanoic and propanoic acids than *Lp. plantarum* LPAL.<br>The highest concentration of hexanoic acid was found in the HC fecal media inoculated with *B. animalis* BLC1.	The resulting best SCFA-producing probiotics chosen to constitute the innovative synbiotic were *B. animalis* BLC1 and *Lc. casei* LC4P1.
Stability test of NatuREN P	• *B. animalis* BLC1 (10^9 cells)<br>• *Lc. casei* LC4P1 (10^9 cells)<br>• FOS (2.5 g)<br>• inulin (2.5 g)<br>• pomegranate seed extract (0.2 g)<br>• maltodextrins from corn (0.5 g)<br>• sodium cyclamate (0.01 g)	The cell density of probiotics underwent a decrease of one or more logarithmic cycles already after 2 months at both room (+20°/+30° C) and refrigerated (+2°/+8° C) temperature.	Rejected
Stability test of NatuREN G	• *B. animalis* BLC1 (10^9 cells)<br>• *Lc. casei* LC4P1 (10^9 cells)<br>• FOS (2.5 g)<br>• inulin (2.5 g)<br>• quercetin (0.064 g)<br>• resveratrol (0.023 g)<br>• grapeseed powder extract (0.013 g) proanthocyanidins (tit. 95% d. extr.)<br>• maltodextrins from corn (0.5 g)<br>• sodium cyclamate (0.01 g)	In NatuREN G, the probiotics' cell density remained approximately stable after two months of product storage at both room (+20°/+30° C) and refrigerated temperature (+2°/+8° C).<br>After 6 months of storage, a slight decrease in cell density was found, but values of cell density were higher than 8 log CFU/g.	Optimal

* (Pom.S.E.): commercial pomegranate seed extract; §(HC-FM): healthy controls- fecal media; $(CKD-FM): chronic kidney disease- fecal media; £(GC-MS): gas-chromatography mass-spectrometry.
