# Supplementary figures and images for: In Vitro Selection of Probiotics, Prebiotics, and Antioxidants to Develop an Innovative Synbiotic (NatuREN G) and Testing Its Effect in Reducing Uremic Toxins in Fecal Batches from CKD Patients
Source: Microorganisms. 2021 Jun 17;9(6):1316. doi: 10.3390/microorganisms9061316 (PMC8235484; doi:10.3390/microorganisms9061316)

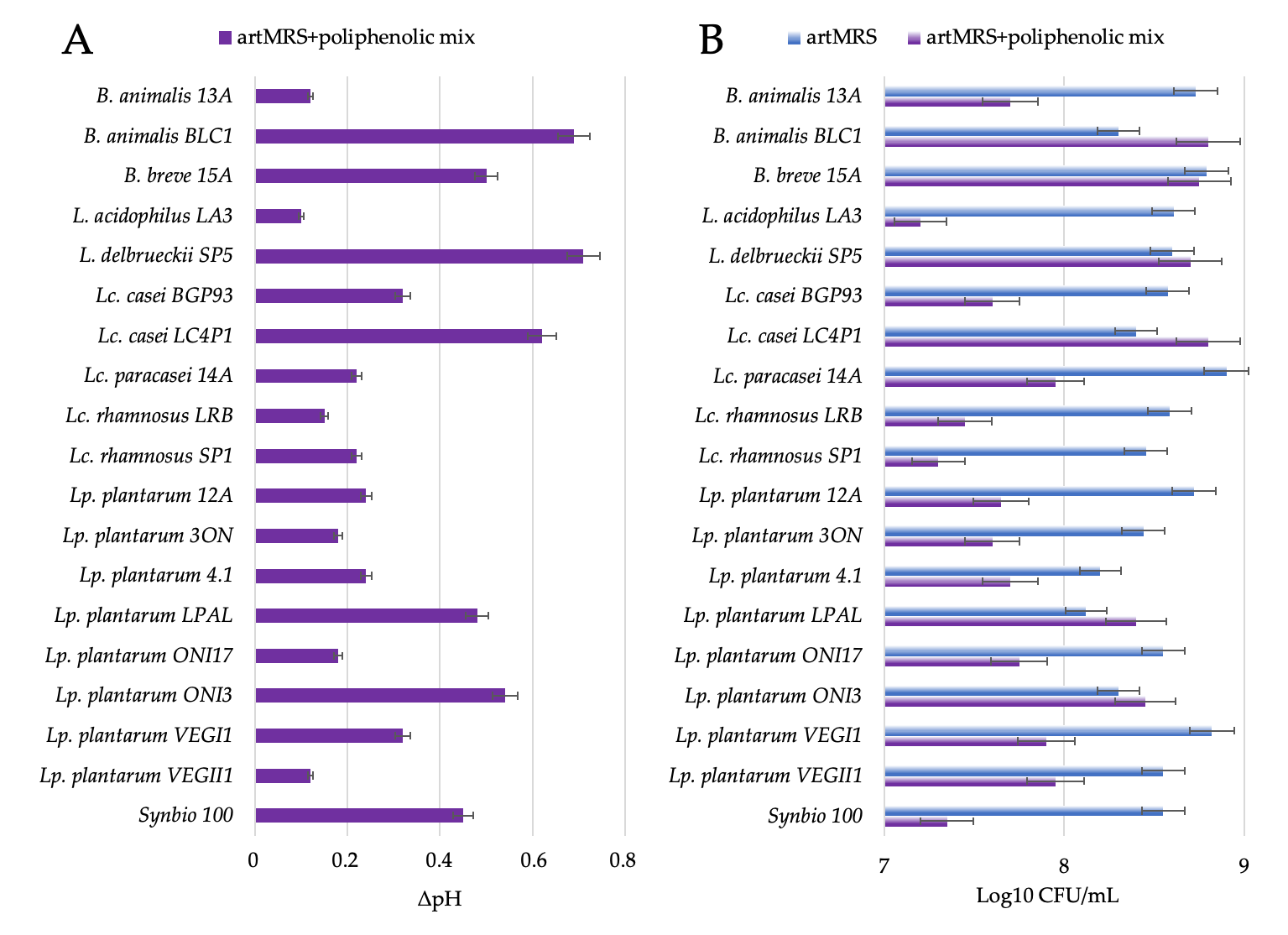

Supplement: Supplementary file 1 [file microorganisms-09-01316-s001.zip › Supplementary_Figure_S1.png]
